# Case Report: Supraventricular Arrhythmia after Exposure to Concentrated Ambient Air Pollution Particles

**DOI:** 10.1289/ehp.1103877

**Published:** 2011-09-06

**Authors:** Andrew J. Ghio, Maryann Bassett, Tracey Montilla, Eugene H. Chung, Candice B. Smith, Wayne E. Cascio, Martha Sue Carraway

**Affiliations:** 1 Environmental Public Health Division, National Health and Environmental Effects Research Laboratory, U.S. Environmental Protection Agency, Chapel Hill, North Carolina, USA; 2 Division of Cardiology, University of North Carolina–Chapel Hill, Chapel Hill, North Carolina, USA

**Keywords:** air pollution, arrhythmias, atrial fibrillation, atrial flutter, heart diseases, particulate matter

## Abstract

Context: Exposure to air pollution can result in the onset of arrhythmias.

Case presentation: We present a case of a 58-year-old woman who volunteered to participate in a controlled exposure to concentrated ambient particles. Twenty minutes into the exposure, telemetry revealed new onset of atrial fibrillation. The exposure was discontinued, and she reverted to normal sinus rhythm approximately 2 hr later. No abnormality was evident on the volunteer’s laboratory examination or echocardiography that could explain an increased risk for supraventricular arrhythmia.

Discussion: Epidemiologic evidence strongly supports a relationship between exposure to air pollutants and cardiovascular disease, but population-level data are not directly relevant to the clinical presentation of individual cases. To our knowledge, this is the only case report of an individual suffering an episode of atrial fibrillation after exposure to an air pollutant. The resolution of the arrhythmia with termination of the particle exposure further supports a causal relationship between the two.

Relevance to clinical practice: Exposure to air pollution, including particulate matter, may cause supraventricular arrhythmias.

Epidemiologic investigation supports a positive relationship between exposure to air pollution and cardiovascular disease (Rich et al. 2006), with the number of deaths from such illness estimated to exceed that for respiratory disease after exposures to elevated levels of pollutants (Dockery 2001). Air pollutants have been associated with acute cardiac events, including myocardial infarctions and cardiac arrests (Forastiere et al. 2005; Rosenthal et al. 2008; Zanobetti and Schwartz 2005). In addition, air pollution has been associated with the incidence of cardiac arrhythmias (Link and Dockery 2010; Peters et al. 2000; Routledge and Ayres 2005). Studies have demonstrated that discharges by implantable cardioverter defibrillators (ICDs) for ventricular arrhythmias increase with higher levels of black carbon, fine particles, coarse particles, nitric oxide, ozone, nitrogen dioxide, nitric oxide, carbon monoxide, and sulfur dioxide (Metzger et al. 2007; Peters et al. 2000; Rich et al. 2005, 2006; Santos et al. 2008). Evidence also supports an association between measures of air pollution and the incidence of supraventricular arrhythmias. In an ICD study, Rich et al. (2005) found a statistically significant relationship between the incidence of supraventricular arrhythmias and increased ozone concentrations in the hour preceding the arrhythmia. Holter examinations revealed an increased risk of supraventricular arrhythmias in association with 5-day moving averages of fine particles ozone, and sulfate in nonsmoking adults (Sarnat et al. 2006). In yet another Holter study, Berger et al. (2006) found that both supraventricular and ventricular arrhythmias increased in association with PM and nitrogen dioxide exposures (in the previous 24–72 hr and with 5-day moving averages) among men with coronary artery disease. These arrhythmias developed within a few hours of increased levels of air pollution (Ljungman et al. 2008).

Although epidemiologic data strongly support a relationship between exposure to air pollutants and cardiovascular disease, this methodology does not permit a description of the clinical presentation in an individual case. To our knowledge, this is the first case report of cardiovascular disease after exposure to elevated concentrations of any air pollutant.

## Case Presentation

A 58-year-old Caucasian female visited the U.S. Environmental Protection Agency’s Human Studies Facility in Chapel Hill, North Carolina, to participate in a study requiring sequential exposures to filtered air and concentrated ambient particles (CAPs). Protocols and consent forms were approved by the University of North Carolina (UNC) School of Medicine Committee on the Protection of the Rights of Human Subjects, and the subject provided informed consent. Two years previously, she had participated in an identical exposure protocol without any complication. At that time, two 24-hr Holter examinations obtained during the exposures to filtered air and CAPs demonstrated 29 and 54 episodes of supraventricular ectopy, respectively.

**Figure 1:**
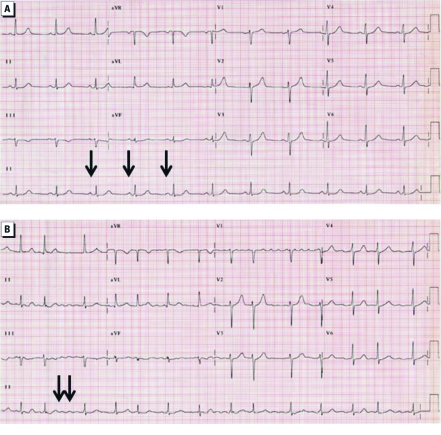
The volunteer’s ECG (12 lead and rhythm strip) before (*A*) and immediately after (*B*) exposure to concentrated ambient particles. The ECG before the exposure (*A*) reveals a regular sinus rhythm with defined P waves (arrows); the ECG after the exposure (*B*) is irregular, with “flutter” waves (arrows).

On the day of exposure to CAPs, the volunteer had no symptoms. The participant had a history of osteoarthritis and hypertension, and she was being treated with an angiotensin-converting enzyme inhibitor and a diuretic (10 mg lisinopril and 12.5 mg hydrochlorothiazide, respectively) for the hypertension. Previous surgeries included a hernia repair, a cholecystectomy, and a total left knee arthroplasty. Her family history was significant due to the death of her father at 57 years of age from a myocardial infarction. The volunteer was a lifetime nonsmoker. On physical examination, she was 173 cm tall and weighed 104.4 kg (body mass index, 34.9; waist, 45 inches). Her pulse was regular at 66/min, and her blood pressure was 144/61 mmHg. The baseline electrocardiogram (ECG) showed normal sinus rhythm (Figure 1A). A Holter monitor demonstrated evidence of increased supraventricular ectopy, with 157 ± 34 premature atrial contractions per hour during the 3 hr immediately preceding the exposure to CAPs.

Twenty-three minutes into the exposure to CAPs (filter weight, 112 μg/m^3^; particle number, 563,912/cc), the telemetry monitor revealed that the subject had nonsustained atrial fibrillation that quickly organized into atrial flutter. She was immediately removed from the exposure chamber. The volunteer reported no symptoms, and there was no change in the physical examination. The 12-lead ECG verified that she remained in atrial flutter (Figure 1B). Her serum electrolytes, blood urea nitrogen, creatinine, glucose, and complete blood count were all normal. Creatine kinase and the MB fraction were also normal. During the transfer to the UNC Medical Center (~ 2 hr after the onset of the arrhythmia), she spontaneously reverted to a normal sinus rhythm.

The patient was admitted to the hospital overnight for observation and telemetry. The next morning, the ECG documented normal sinus rhythm. Her serum electrolytes, blood urea nitrogen, creatinine, glucose, creatine kinase, and the MB fraction were again normal, and her complete blood count was normal except for a hematocrit of 35.7% (the lower limit of normal is 36.0%). Resting transthoracic echocardiography demonstrated normal right ventricular contraction with an ejection fraction of 55–60%, aortic sclerosis, and diastolic left ventricular dysfunction. The left atrium was considered mildly dilated; all other chambers of the heart were normal in size. She was discharged and was not prescribed a new medication. Approximately 6 weeks later, she underwent electrophysiology study, which did not provoke atrial fibrillation or significant atrial ectopy. The study did indicate a reentrant circuit of the cavotricuspid isthmus, which was ablated to prevent potential future episodes of atrial flutter.

## Discussion

The volunteer demonstrated evidence of increased supraventricular ectopy immediately preceding her exposure to CAPs, but there was no evidence of atrial arrhythmias. She then suffered the onset of atrial fibrillation a very short time after being exposed to CAPs. Within 2–3 hr after the exposure stopped, the arrhythmia resolved and she returned to normal sinus rhythm. Atrial fibrillation is the most common supraventricular arrhythmia, affecting 1–2% of the general population (Falk 2001). This arrhythmia is uncommon in people < 60 years of age, but it afflicts about 10% of the population by 80 years of age. Risk factors for atrial fibrillation include hypertension (especially uncontrolled), coronary artery disease, heart failure, cerebrovascular disease, diabetes, thyroid conditions, sleep apnea, obesity, a past history of rheumatic heart disease and congenital heart defects, pericarditis, sick sinus syndrome, a family history of atrial fibrillation, and echocardiographic abnormalities (Kannel and Benjamin 2008, 2009). In addition, cigarette smoking, alcohol use, caffeine consumption, and stimulant drugs can help trigger atrial fibrillation. Of these defined risk factors, the volunteer had a history of well-controlled hypertension, and her body mass index was consistent with obesity. Her history of premature atrial contractions may also have increased her risk for atrial fibrillation (Binici et al. 2010). In a similar manner, preexisting cardiovascular disease, diabetes and impaired glucose tolerance, chronic obstructive pulmonary disease, and current cigarette smoking all increase susceptibility for cardiovascular disease associated with air pollution (Chen et al. 2006; Liao et al. 2009; Mills et al. 2007; Wheeler et al. 2006; Whitsel et al. 2009; Zareba et al. 2009). There was no obvious explanation for her onset of a supraventricular arrhythmia during the exposure. Although coincident atrial fibrillation cannot be excluded, the onset of her arrhythmia was associated with her exposure to ambient air pollution particles. The correlation between the resolution of the arrhythmia and the termination of the CAP exposure further supports a causal relationship between the two.

Systemic inflammation and underlying oxidative stress may increase the risk of atrial fibrillation (Kumagai et al. 2004). Patients with atrial fibrillation demonstrate evidence of inflammation, with elevated levels of inflammatory markers, including C-reactive protein, interleukin-6, and tumor necrosis factor-α (Chung et al. 2001; Gaudino et al. 2003). Some evidence suggests that statin treatment may potentially alter the risk for this arrhythmia by modifying oxidative stress (Siu et al. 2003). The specific association between increased arrhythmia induction and air pollution may reflect oxidant generation and inflammation after exposure, consistent with mechanisms involved in the initiation and maintenance of some other forms of atrial fibrillation (Mazzoli-Rocha et al. 2010). The oxidative stress and inflammation associated with the pollutant have been postulated to affect coronary perfusion and consequently enhance the propensity for such arrhythmias through tissue ischemia. However, the rapid onset of this volunteer’s atrial fibrillation after CAP exposure suggests that the basis for the arrhythmia may be a disruption of the normal cardiac autonomic control rather than a systemic inflammation, because the latter would require a longer period of time to develop (Routledge and Ayres 2005). In an animal model, diesel exhaust increased the sensitivity of the heart to triggered arrhythmias via an activation of airway sensory receptors [e.g., TRPA1 (transient receptor potential cation channel A1)] (Hazari et al. 2011). Several researchers have suggested that this leads to autonomic imbalance and a predisposition for arrhythmia development. A comparable mechanism has been proposed to explain the cardiac response to ozone and cigarette smoke (Joad et al. 1998; Mutoh et al. 2000).
